# The application of compressive sampling in rapid ultrasonic computerized tomography (UCT) technique of steel tube slab (STS)

**DOI:** 10.1371/journal.pone.0190281

**Published:** 2018-01-02

**Authors:** Baofeng Jiang, Pengjiao Jia, Wen Zhao, Wentao Wang

**Affiliations:** 1 College of Resource and Civil Engineering, Northeastern University, Shenyang, Liaoning Province, China; 2 Department of Civil and Environmental Engineering, University of Michigan, Ann Arbor, MI, 48109–2125, United States of America; China University of Mining and Technology, CHINA

## Abstract

This paper explores a new method for rapid structural damage inspection of steel tube slab (STS) structures along randomly measured paths based on a combination of compressive sampling (CS) and ultrasonic computerized tomography (UCT). In the measurement stage, using fewer randomly selected paths rather than the whole measurement net is proposed to detect the underlying damage of a concrete-filled steel tube. In the imaging stage, the ℓ1-minimization algorithm is employed to recover the information of the microstructures based on the measurement data related to the internal situation of the STS structure. A numerical concrete tube model, with the various level of damage, was studied to demonstrate the performance of the rapid UCT technique. Real-world concrete-filled steel tubes in the Shenyang Metro stations were detected using the proposed UCT technique in a CS framework. Both the numerical and experimental results show the rapid UCT technique has the capability of damage detection in an STS structure with a high level of accuracy and with fewer required measurements, which is more convenient and efficient than the traditional UCT technique.

## Introduction

Recently, transit infrastructure condition assessment has become an important subject attracting researchers’ attention. The need for better methods of nondestructive evaluation to inspect the structural damage of infrastructures is increasing all over the world [1–3]. A significant challenge for engineers and managers is the nondestructive evaluation of subway construction projects that located in complex environments, *e*.*g*., under existing buildings or underground crossings of major municipal arteries and pipelines [4–6]. Such non-excavation projects need more concise and rapid detection methods.

After years of research and application, the traditional shallow tunneling method (STM), which was commonly used to construct the urban subway tunnel in China has developed into an innovative design method for underground structures using the steel tube slab (STS) structure without temporary support during the excavation [7, 8]. It has the ability to improve the transverse load-carrying capacity by adding transverse high strength bolts and flange plates between the tubes [9]. The STS structure serves as a primary support that resists the overburdening earth pressure and ground overload during excavation. The maximum transverse span of the STS structure is 5m during excavation.

Shenyang Metro line 9’s Olympic Center station and line 10’s Northeastern Street station are both super shallow buried stations (soil cover depth less than 5 m), located downtown. To overcome the difficulties, STS structure is proposed to construct this subway station. Its load-carrying capacity is an essential part of the entire project, while the absence of concrete in the tubes is the weakest part of the STS structure, which is subjected to flexural and shear effects. Thus, before the service stage, the investigations of the flaws and holes in the concrete must be completed using ultrasonic computerized tomography (UCT) to diagnose the internal situation of the concrete-filled tubes to predict the flexural capacity of the STS structure.

UCT is a non-destructive ultrasonic testing technique containing two stages: 1) the measurement stage and 2) the digital imaging stage. In the measurement stage, the transducer-receiver pair physically rotates or rearranges around the object to sequentially provide multiple measurements of all elements. Each measurement is enclosed by ultrasonic characteristics, such as the speed of sound, mass density and attenuation [10]. Meanwhile, in the imaging stage, computerized tomography provides the quantitative information of the monitored structure based on the inverse scattering algorithm [11–13].

UCT has been extensively developed and applied in many areas, such as medicine, material engineering, aerospace, civil engineering. Greenleaf and Bahn developed a UCT technique to diagnose breast cancer. The measured arrival time and changes in amplitude were used in a reconstruction algorithm to obtain estimates of the 2-D distribution of acoustic speed and attenuation of the breast. The quality of over 1000 scanned images demonstrating the sensitivity of the UCT system connects to the work done by using the X-ray technique [11]. Rahiman et al. obtained the mode tomography imaging for liquid/gas two-phase flow by using the UCT technique [14]. Hay et al. used Lamb waves ultrasonic computerized tomography to identify the material loss of real aircraft components by embedding piezoelectric sensors on the surfaces [15]. Büyüköztürk imaged a complex concrete structure via UCT, indicating this technique has a high level of accuracy at resolution [16]. Bond et al. used acoustic travel time tomography to assess a mass concrete dam presenting cross-sectional images of the structure to locate cracks and other damage [17, 18].

In this paper, reconstruction of the element’s slowness (speed) has been exploited for the purpose of guaranteeing the safety of STS structures. However, detection accuracy needs hundreds of measurement paths, which makes the damage detection process an enormous task and limits the application of the traditional UCT technique [19, 20]. Therefore, a rapid UCT technique that can reduce both measurement numbers and computational cost is proposed based on the compressive sampling (CS) theory. van Sloun et al. proposed a CS solution for UCT to adopt a measurement scheme based on compressed acquisitions [20]. Reconstruction of the image was obtained by combining the born iterative method and total variation minimization, thereby exploiting variation sparsity in the image domain. Simulated UCT scattering measurements showed their proposed method was better than the uniform under-sampling method, with a drastic reduction of acquisition time. Hua et al. presented a reconstruction method based on CS for sparse-view ultrasonic diffraction tomography [21]. Their numerical experiments showed that their proposed method was effective in improving image quality with the same number of views. Huy et al. proposed a deterministic measurement method in the detection geometry and image reconstruction process using ℓ1-regularization [22]. Their simulation results demonstrated high performance, with half the measurements used in the traditional method and only two iterations. However, their research lacks application in real-world structures, which are much more complex than simulation results.

This paper focuses on the rapid UCT method based on an algorithm of compressive sampling for the damage detection of STS structures in the Shenyang Metro station. Firstly, the UCT technique and the basic formula are introduced briefly in section 2. Then, in section 3, a new compressive sampling algorithm is applied for the reconstruction of UCT, and the whole conventional UCT program is improved and redesigned to reduce the number of measurements required for damage detection. In section 4, three different numerical models with various levels of damage are studied to validate the proposed algorithm. And in section 5, experimental damage detection testing of real-world concrete-filled steel tubes in the Shenyang Metro stations are implemented to represent the capability of the improved UCT technique. Conclusions and the scope for future work are discussed in the final section.

## Ultrasonic computerized tomography

**Fig 1:**
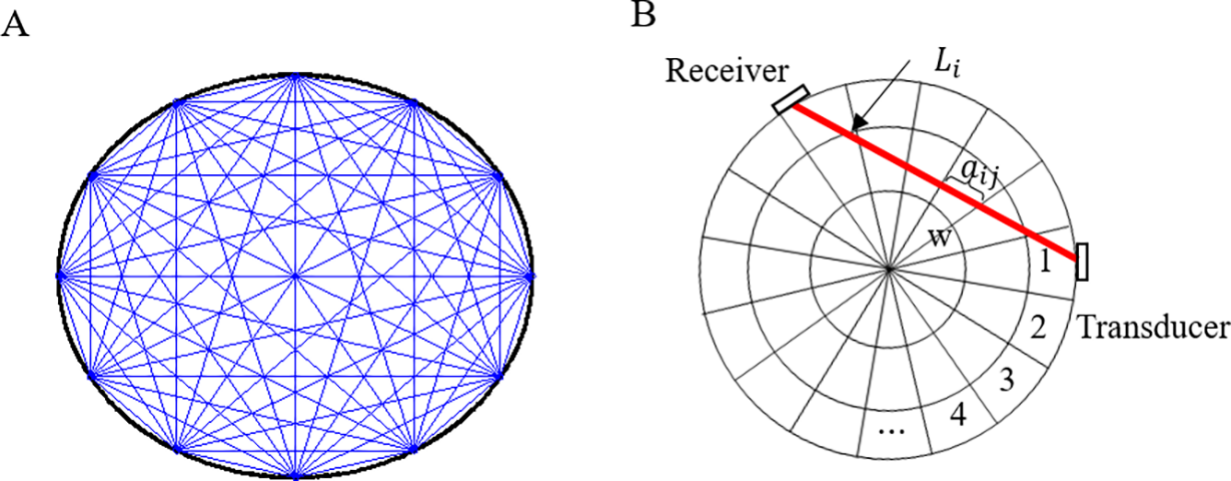
Schematic of UCT of an STS concrete-filled tube. (A) Measurement net. (B) Measurement path and transducer-receiver pair.

Ultrasonic computerized tomography is very similar to X-ray tomography, with the ability to illuminate and estimate the internal areas by measuring the energy or traveling time on the outside of the object. Compared to X-ray, ultrasonic eliminates the drawback of exposing the technician to a small dose of repeated radiation. In UCT, it is essential to have a dense net of various measurement paths that ultrasonic waves traverse from transducers to receivers, as shown in Fig 1. To reconstruct internal damage, the monitored structure is discretized into numerous microstructures. Measured points for transducers and receivers are located on the outer surface to introduce and sense the ultrasonic waves. Acoustic parameters of the ultrasonic waves including velocity, amplitude, and attenuation are evidence a damaged microstructure. Generally speaking, ultrasonic waves propagate at high speed on a compacted area, and at low speed on a damaged part of an object. This is because the defects, such as holes or flaws, cause diffraction, causing the wave to have to cross the edge via a longer path to the receiver. In this case, the UCT is computed by taking multiple scans at various rotations around the STS structure being imaged. The transducer-receiver coupled arrays physically rotate around the object, giving multiple pathways through the body. Based on the density of areas within the object, the travel time of the ultrasonic wave is delayed at different levels. This results in a signal of the projection of the image with attributes only on the measurement path that interacts with the damaged microstructure. Multiple measurements carry the information of both healthy and damaged microstructures. Thus, the internal situation of the monitored structure can be reconstructed by external measurements.

Basic UCT formulas are shown as follows. The variation from the expected travel time is attributed to inhomogeneity in the structure or to damage occurred. To pursue an accurately reconstructed result, the monitored structure has to be adequately covered by a dense net consisting of multiple measurement paths, as shown in Fig 1.

The travel time in a selected path is witnessed to change with both the travel path and the microstructures, which can be expressed as:

$$T_{i}=\int_{L_{i}}\frac{1}{V(x,y)}dL=\int_{L_{i}}s(x,y)dL$$ (1)

where *T*_*i*_ is the travel time of the *i*^*th*^ measuring path, *L*_*i*_ is the distance of the *i*^*th*^ measurement path from the transducer to the receiver, *V*(*x*,*y*) and *s*(*x*,*y*) are the velocity function and slowness function of the microstructure at location (*x*,*y*).

As previously proposed, the monitored structure is discretized into numerous microstructures. Thus, the measured travel time ***T*** = (*T*_1_,*T*_2_,⋯,*T*_*N*_)^*T*^ can be expressed as Eq (2), which combines the travel time of all the measurement paths:

$$\left\{ \begin{array}{l} T_{1}=\sum_{j=1}^{w}\frac{a_{1j}}{V_{j}}=\frac{a_{11}}{V_{1}}+\frac{a_{12}}{V_{2}}+\cdots +\frac{a_{1w}}{V_{w}}=a_{11}s_{1}+a_{12}s_{2}+\cdots +a_{1w}s_{w}\\ T_{2}=\sum_{j=1}^{w}\frac{a_{2j}}{V_{j}}=\frac{a_{21}}{V_{1}}+\frac{a_{22}}{V_{2}}+\cdots +\frac{a_{2w}}{V_{w}}=a_{21}s_{1}+a_{22}s_{2}+\cdots +a_{2w}s_{w}\\ \;\vdots \\ T_{n}=\sum_{j=1}^{w}\frac{a_{nj}}{V_{j}}=\frac{a_{n1}}{V_{1}}+\frac{a_{n2}}{V_{2}}+\cdots +\frac{a_{nw}}{V_{w}}=a_{n1}s_{1}+a_{n2}s_{2}+\cdots +a_{nw}s_{w} \end{array}\right.$$ (2)

$$T=A∙\frac{1}{V}=A∙s=A∙(s_{0}+\Delta s)$$ (3)

where *w* is the total number of microstructures, *N* is the number of measurement paths, *T*_*i*_ is the travel time of ultrasonic waves along the *i*^*th*^ measurement path, *a*_*ij*_ is the travel length of the *j*^*th*^ microstructure along the measurement *i*^*th*^ path, ***A*** is the matrix of travel length of all the microstructures, *V*_*j*_ is the velocity of the ultrasonic waves propagating in the *j*^*th*^ microstructure, *s*_*j*_ is the slowness of the *j*^*th*^ microstructure, ***s*** is the vector of the slowness of all the microstructures in the section, ***s***_**0**_ is the standard slowness of ultrasonic waves propagating in the structure, and Δ***s*** is the difference of the slowness.

After the measurement stage, inversion algorithms are employed for the tomographic reconstruction, such as algebraic reconstruction techniques (ART), simultaneous iterative reconstruction techniques (SIRT), least squares QR-factorization (LSQR), etc. The flaws and holes have the effect of increasing the residual of tomographic velocity reconstruction and lead to an identification of false anomalies. The accuracy of the slowness reconstruction can be improved by a better understanding of the measurement signals and a planned dense net of measurement paths, including the choices of position and number of the sensing station.

## Compressive sampling algorithm

Conventional reconstruction algorithms require a dense net of measurement paths that contain many redundant paths. In this paper, a novel reconstructed algorithm based on compressive sampling is proposed to reduce the number of measurements and to speed up damage detection processing. Compressive sampling is a new theory of information acquisition proposed by D. Donoho [23], E. Candès [24] and T. Tao et al. [25] in 2006. It asserts that one sparse signal can be acquired and recovered from considerably fewer linear measurements than the Shannon/Nyquist theorem suggests. Assuming there is a discrete time signal **x** ∈ ℝ^*N*^, which can be expressed as a K-sparse (i.e., with K<< N nonzero entries) vector in a certain basis, and the unknown signal **x** is defined to be compressible when it can be expressed as a K-sparse vector ***α*** ∈ ℝ^*N*^ (i.e., it has only *K* ≪ *N* nonzero entries) with respect to a specific basis **Ψ** ∈ ℝ^*N*×*N*^, expressed as:

x=Ψα (4)

where **Ψ** ∈ ℝ^*N*×*N*^ is the basis matrix and **α** ∈ ℝ^*N*^ is a K-sparse vector. The above signal can be captured through a small number m (*K* < *m* ≪ *N*) of the measurement sample vector **y** ∈ ℝ^*m*^. In addition, considering measurement noise, the vector **y** can be expressed as follows:

y=ΦΨα+n=Θα+n (5)

where **Φ** ∈ ℝ^*m*×*N*^ is the measurement matrix, ***n*** denotes the noise during the measuring process, **Θ** = **ΦΨ** is defined as the transfer matrix, and the rows of matrix **Θ** are much fewer than the columns. Because the number of measurements (the dimension of **y**) is smaller than the number of samples in the original signal (the dimension of **x**), solving of the original signal from measurements becomes an ill-posed inverse problem with infinite solutions [26, 27]. However, an approximate recovery is possible by solving a convex optimization problem when the original signal **x** is sparse within a specific basis [24] (e.g., frequency basis and wavelet basis).

Moreover, the following restricted isometry property (RIP) of the transfer matrix **Θ** is required to reconstruct the unknown signal without loss of information [23]:

$$1-\delta \leq \frac{{\left\|\Theta v\right\|}_{2}}{{\left\|v\right\|}_{2}}\leq 1+\delta ,\delta >0$$ (6)

where, **v** ∈ ℝ^*N*^ is all *K*-sparse vector and *δ* is the smallest value that satisfies Eq (6) [23]. In summary, CS is a theoretical framework relying on sparsity and incoherence (RIP of the transfer matrix). The smallest number of required measurements is *m* > *μ* ∙ *K* ∙ log(*N*/*K*), where *μ* is a constant (in general, *μ* = 4) when both the RIP and incoherence are achieved by selecting **Θ** as a random matrix (Gaussian matrix or Bernoulli matrix).

Thus, there are three requirements: 1) the *K*-sparse signal ***α*** in a specific basis, 2) the RIP of the transfer matrix **Θ** = **ΦΨ**, and 3) the sufficient numbers of linear measurements *m* > *μ* ∙ *K* ∙ log(*N*/*K*). Then, the original signal **x** can be exactly or approximately reconstructed from the random low-rate sampling measurement vector **y** by solving a convex program via the *l*_1_-norm optimization algorithm:

$$\min\;{\left\|\alpha \right\|}_{1}\;subject\;to\;{\left\|\Theta \alpha -y\right\|}_{2}<\varepsilon$$ (7)

where ${\left\|\alpha \right\|}_{1}=\sum_{i=0}^{N}\left|\alpha_{i}\right|,\;{\left\|∙\right\|}_{1}$ is the *l*_1_-norm, ‖∙‖_2_ is the *l*_2_-norm, and *ε* is the noise bound.

**Fig 2:**
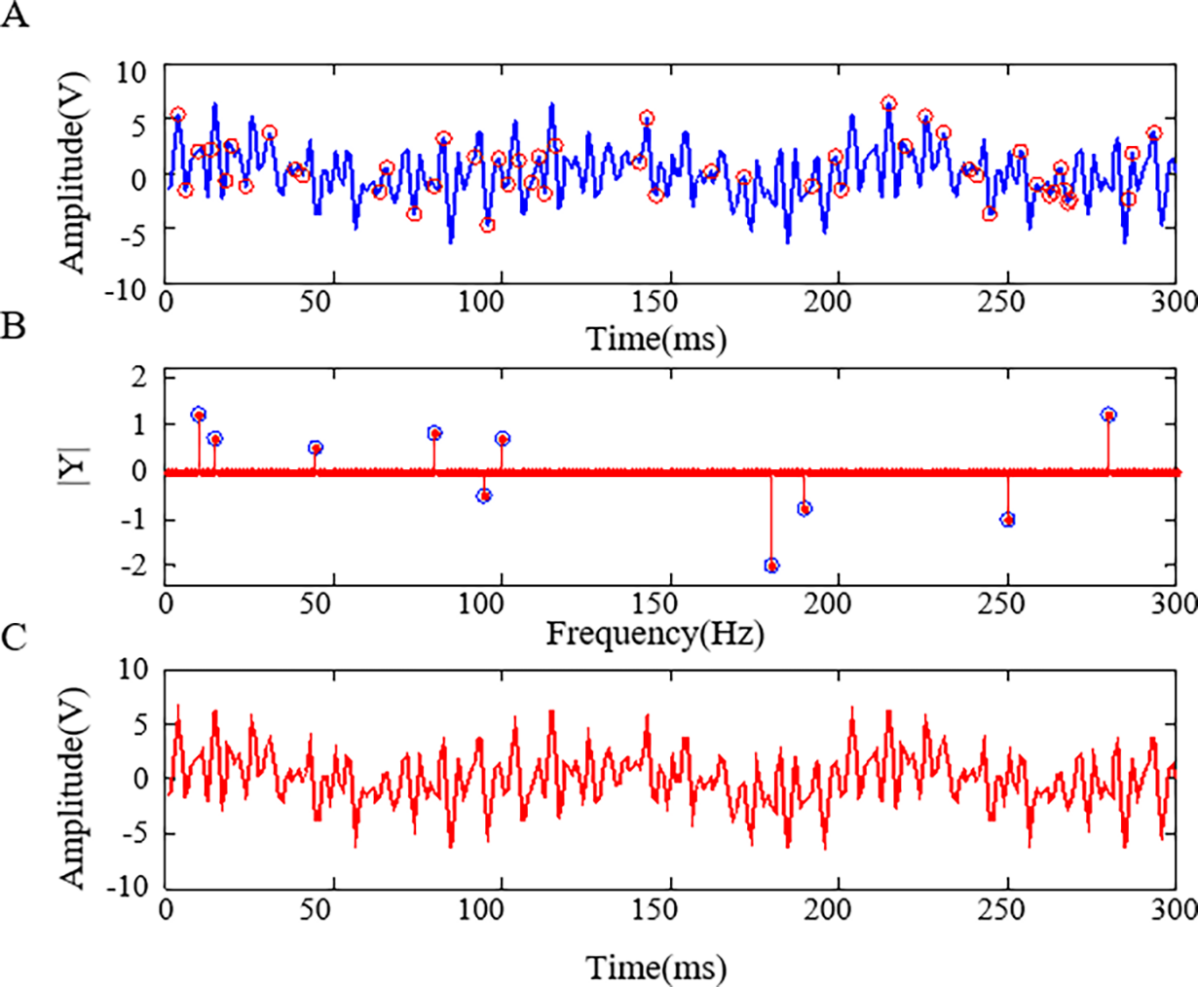
Compressive sampling and recovering of an example signal sparse in the frequency domain. (A) Original signal and the random sampling points. (B) Original and reconstructed coefficients in the frequency domain. (C) Reconstructed signal based on the sparse coefficients.

To address these concerns, a simple example of the signal captured based on CS theory is shown in Fig 2. Different from conventional Nyquist uniform sampling, CS enables non-uniform low-rate random sampling (based on the Bernoulli distributed matrix, shown as the red circle points in Fig 2A) to capture sufficient information of the example signal. If the underlying signal is sparse in a proper representation domain like the coefficients of the example signal in the frequency domain, then, the random points captured in the sampling stage can be recovered in exactly the same way as the original by using the ℓ1-minimization algorithm. It can be noted that the reconstructed coefficients (red points) calculated by the ℓ1-minimization algorithm match highly the original coefficients (blue circles) of the example signal in the frequency domain as shown in Fig 2B). Finally, the signal is recovered based on the reconstructed coefficients, as shown in Fig 2C). The example signal has 300 points in this period. However, 30 random points captured by the CS method are sufficient for the reconstruction, which represents a compression of the redundant information.

The flaws and holes are naturally sparse-distributed in the structure (sparse in the spatial domain) making the combination of CS and UCT technique possible. Thus, the UCT technique is proposed for execution in the CS framework to simplify the dense measurement net. The sparse representation of the slowness is obtained by the difference Δ***s*** between the slowness of sequential microstructures and standard slowness *s*_0_.

A random Bernoulli distributed matrix **Φ** is proposed to select the measurement paths in the measurement stage. The acquired travel time in the CS framework can be expressed as:

$$T_{m}=\Phi ∙T=\Phi ∙A∙(s_{0}+\Delta s)$$

$$y=\Phi ∙(T-T_{0})=\Phi ∙A∙\Delta s$$ (8)

where, ***T***_***m***_ is the travel time vector acquired by the selected paths, **Φ** is a random Bernoulli distributed matrix related to the selected paths, ***A*** is the matrix of travel length of all the microstructures, ***s***_**0**_ and Δ***s*** are the standard slowness and the slowness difference vector, ***T***_**0**_ is the standard travel time, **y** is the difference of the measured travel time and the standard travel time.

As shown in Eq (9), the random matrix is determined by the Bernoulli function, which contains only 0 or 1. “1” means the related real physics path is selected to measure the travel time in the measurement stage. In contrast, “0” means the real physics path is left out of the measurement net.

$$\Phi ={\left[\begin{array}{cc} \begin{array}{cc} 1 & 0\\ 0 & 0 \end{array} & \begin{array}{ccc} 0 & \cdots & 0\\ 1 & \cdots & 0 \end{array}\\ \begin{array}{cc} 0 & 1\\ \vdots & \vdots \\ 0 & 0 \end{array} & \begin{array}{ccc} 0 & \cdots & 0\\ \vdots & \ddots & \vdots \\ 0 & \cdots & 1 \end{array} \end{array}\right]}_{m\times N}$$ (9)

In summary, because of the naturally sparse distribution of the damaged microstructures, the reconstruction of UCT imaging can be achieved by using a random sampling procedure in a CS framework to speed up the data acquisition stage. With enough measurements *m* > *μ* ∙ *K* ∙ log(*N*/*K*), the proposed ℓ1-minimization algorithm of CS theory is employed to recover Δ***s***, which represents the internal situation of concrete-filled steel tube of the STS structure:

$$\min\;{\left\|\Delta s\right\|}_{1}\;subject\;to\;{\left\|\Theta \Delta s-y\right\|}_{2}<\varepsilon \;where\;\Theta =\Phi A$$ (10)

The sparsest solution of the slowness vector Δs is a physical-meaning solution of the damage detection problem. As a convex optimization problem, the analytical method, the semi-analytical method, and the quantitative method could be used to pursue the sparsest solution in Eq (7) and Eq (10) [28–30]. In this paper, the sparsest solution is solved by the ℓ1-minimization optimization algorithm as a convex optimization toolbox embedded in MATLAB (available at: http://cvxr.com/cvx/). It should be noted that the given CS algorithm would be more effective if a Lagrange multiplier was used in the imaging stage [31, 32]; thereby, Eq (10) will change to Eq (11):

$$\Delta \hat{s}=\mathrm{argmin}\left({\left\|\Theta \Delta s-y\right\|}_{2}++\lambda {\left\|\Delta s\right\|}_{1}\right)\;where\;\Theta =\Phi A$$ (11)

where $\Delta \hat{s}$ is the reconstructed slowness and λ is the Lagrange multiplier, which balances the ratio between the data misfit (‖ΘΔs − y‖_2_) and the model constraint (‖Δs‖_1_) in the optimization equation.

**Fig 3:**
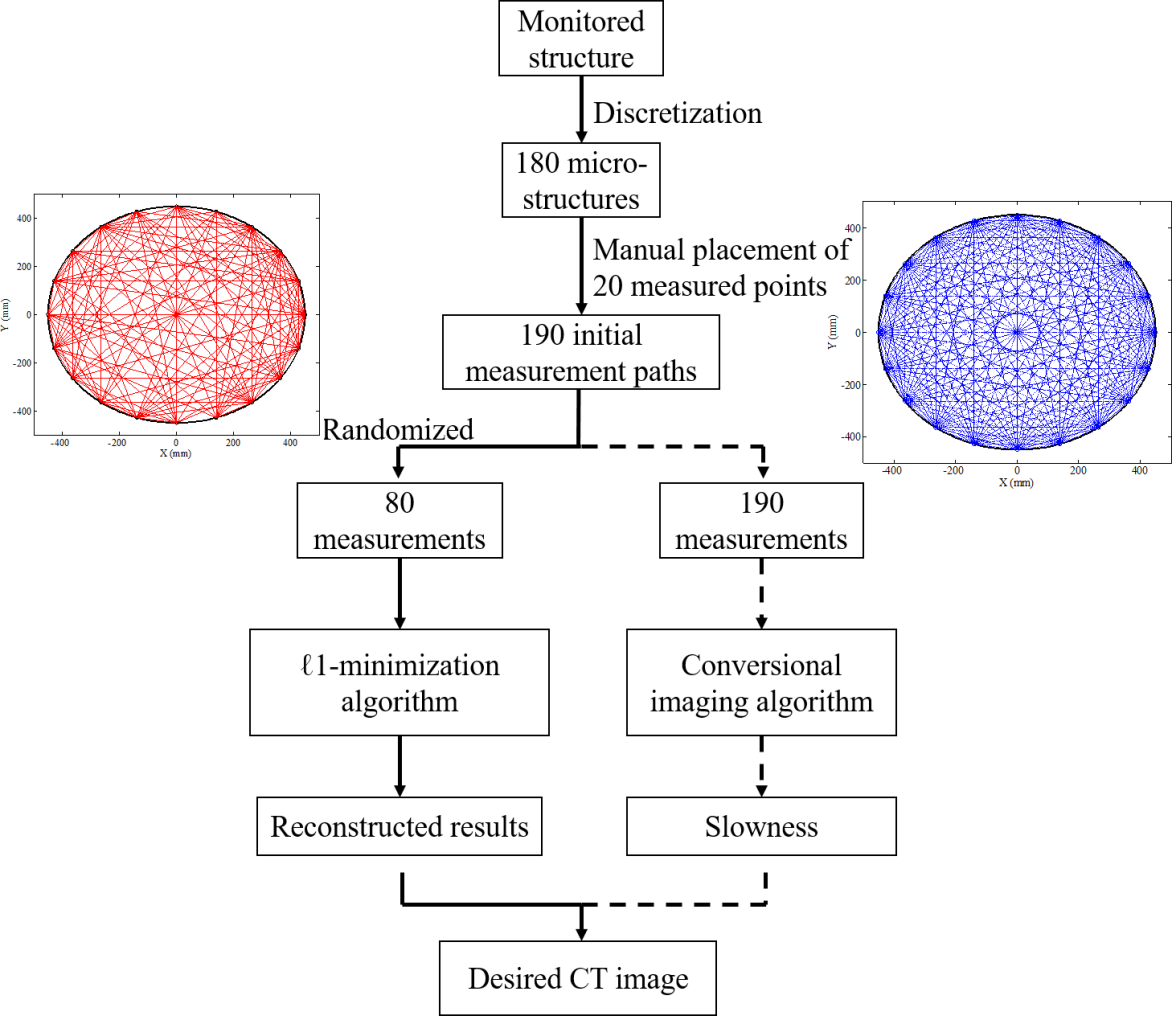
Flowchart of rapid UCT imaging method based in the CS platform.

Fig 3 shows a flowchart of a comparison of the proposed method and conventional UCT: 1) after the discretion of the monitored structure, measurement points, and initial measurement paths are set up on the external shell of the STS structure, 2) random paths are selected based on the Bernoulli distributed matrix to carry out the ultrasonic scanning instead of using the whole measurement net of the conventional UCT technique, 3) the travel time detected by the transducer-receiver pairs is assembled to a vector based on the random matrix for imaging of the concrete-filled steel tube, 4) ℓ1-minimization algorithm of CS theory is employed to recovery the slowness value of each microstructure, which is evidence of the internal situation of the monitored STS structure.

## Numerical validation

**Fig 4:**
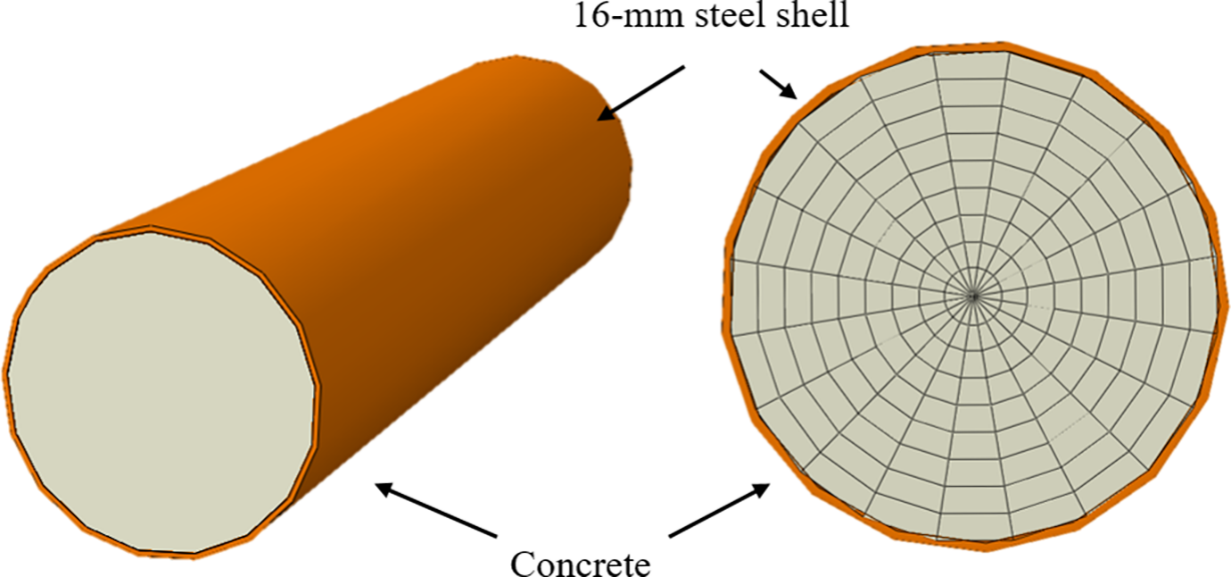
Overall view and microstructures of the concrete-filled steel tube model of STS structure.

Numerical simulations were carried out in ABAQUS, a finite element (FE) software. The models were established based on the real size of the STS structure at the Shenyang Metro’s stations, which is shown in Fig 4. The velocity of the ultrasonic wave propagating in the tube was 4200 m/s, which defines the standard slowness as well. Three different levels of damage were studied to mimic the situation in the real world: 1) healthy case, 2) single-damage case, and 3) multi-damage case. The diameter of the steel tube was 900 mm with a steel-shell thickness of 16 mm. To detect the flaws and holes of the concrete in the tube, the entire tube was divided into 180 microstructures. 20 measurement points and 190 (20 × 19/2) measurements are required for the traditional UCT technique to ensure the specimen passes inspection. Each initial measurement point is represented as a small circle on the external shell, and the measurement paths are shown on the right-hand side of Fig 3. However, based on the compressive sampling algorithm, 80 measurement paths were chosen, which is larger than the minimum number of detections (*M* > *μ* ∙ *K* ∙ log(*N*/*K*) = 58.09) and is sufficient to obtain accurate detection results. The measurement net including the 80 paths selected by the Bernoulli distributed matrix is shown in Fig 3. All the three damage cases are measured by using the same selected measurement net to detect the damage in the models.

### Healthy model

**Fig 5:**
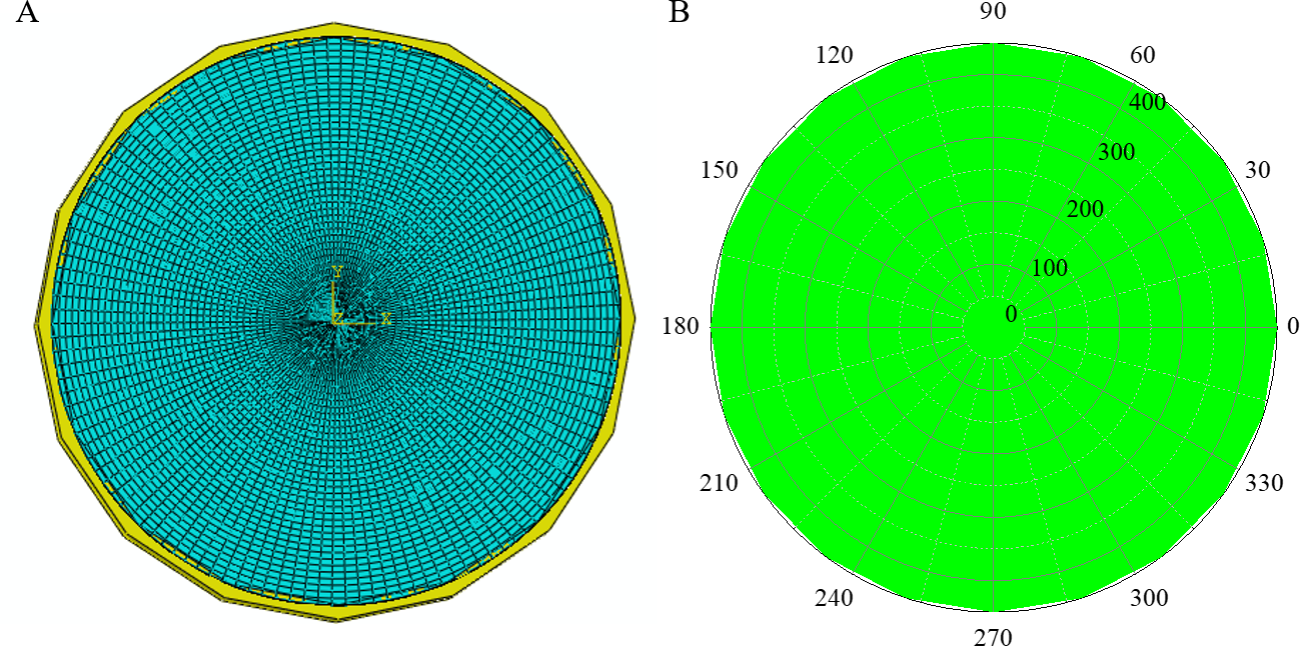
Reconstructed result of healthy model. (A) Cross section of the healthy model. (B) Reconstructed result.

A healthy model of a concrete-filled steel tube based on the real size of the STS structure was established in ABAQUS (Fig 5). The ultrasonic travel time obtained from the numerical model was the same as the standard travel time in the same measurement path. Thus, the difference of the travel time, Δ***T***, is a full-zero vector, which indicates the microstructures are all healthy in this case. It is should be noted that this case is calculated as a comparison condition for the following damaged cases.

### Single-damage case

**Fig 6:**
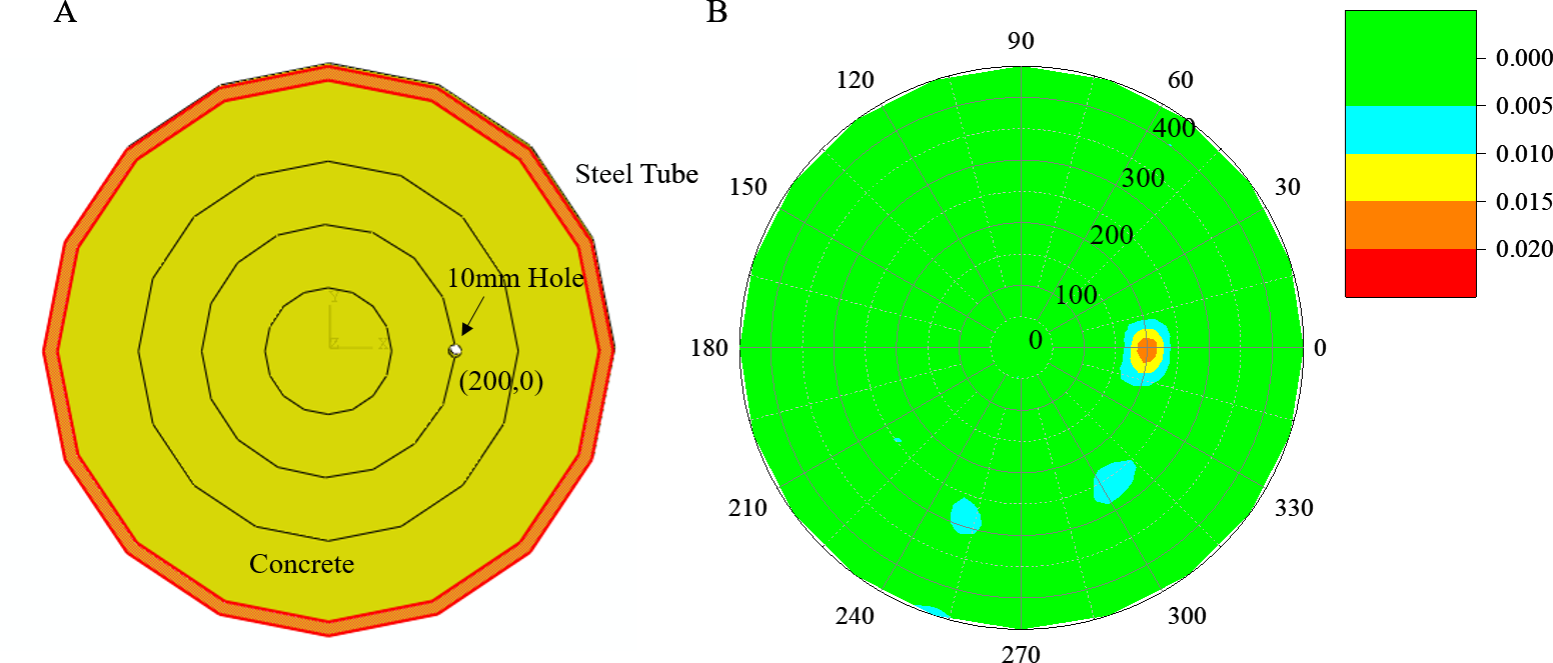
Damage detection of the numerical model with single damage. (A) Section sketch of a concrete-filled steel tube. (B) Reconstructed results.

For the single-damage case, a hole with a diameter of 10 mm was set at location (200,0°) in the polar coordinate system. Based on the Huygens principle, the travel length of ultrasonic waves is curved by the damage resulting in the travel time being longer than the standard time calculated by the pristine tube along the same path. Parts of the measurement paths related to the damage were affected by the hole. Thus, the imaging result calculated by the proposed CS algorithm reveals the location of the damage as shown in Fig 6B). As we can see, the reconstructed damage location is in agreement with the original flaw.

### Multi-damage case

**Fig 7:**
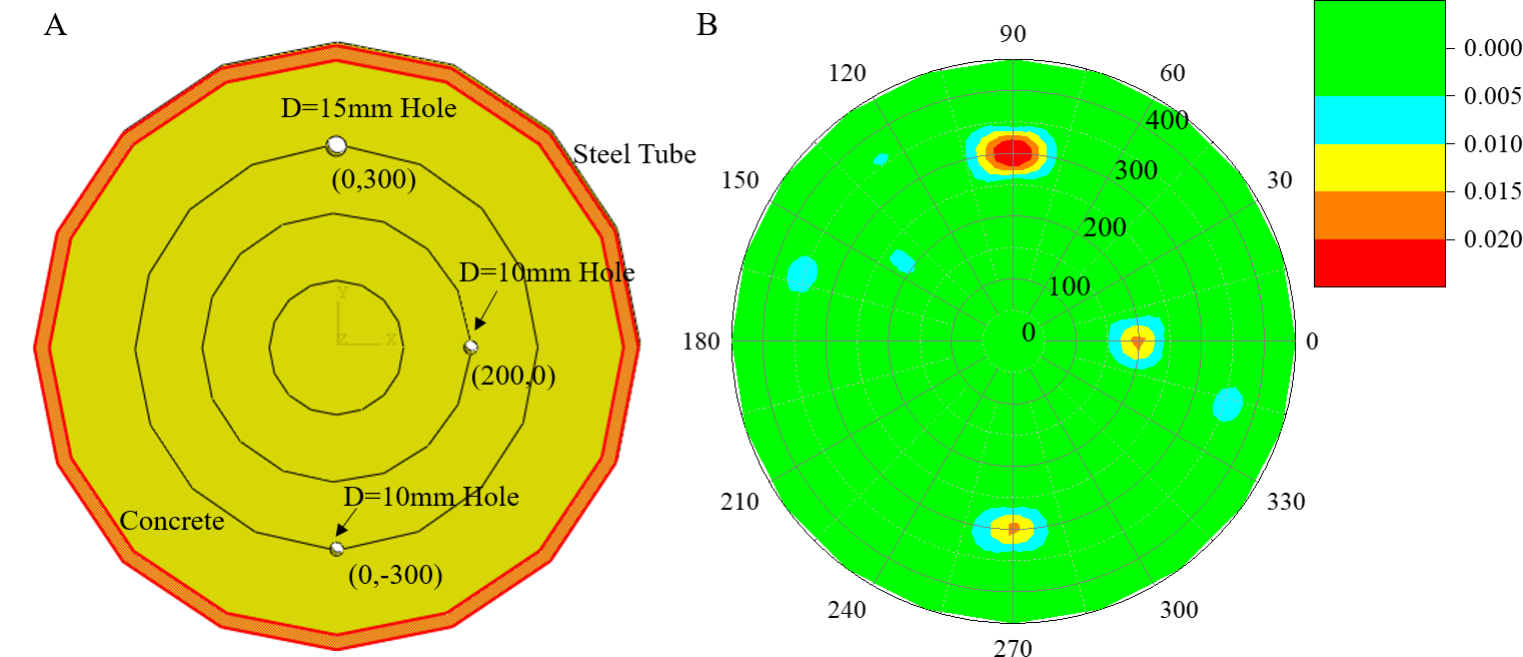
Damage detection of the numerical model with multiple damages. (A) Section sketch of a concrete-filled steel tube. (B) Reconstructed results.

In the multi-damage case, 3 holes were arranged at locations (200,0°), (300,90°) and (300,270°). The diameter of the hole at location (300,90°) is 15 mm, with the other two holes being 10 mm. The section sketch of concrete-filled steel tube model is shown in Fig 7A). As mentioned above, the measurement paths passing along the flaws will cause a longer travel length and a longer travel time of propagating ultrasonic waves. Thus, the damage locations were reconstructed based on the ℓ1-minimization algorithm and CS theory, which is shown in Fig 7B).

All three numerical models indicate that the algorithm is able to reconstruct the damage (holes and flaws) with high level of accuracy in the STS structure concreted-filled tube and with much fewer low-rate measurement paths than required by traditional UCT. Thus, the proposed UCT damage detection method based on CS improves the traditional UCT technique with a high level of accuracy of reconstruction and with fewer required measurements.

## Experiments and results

**Fig 8:**
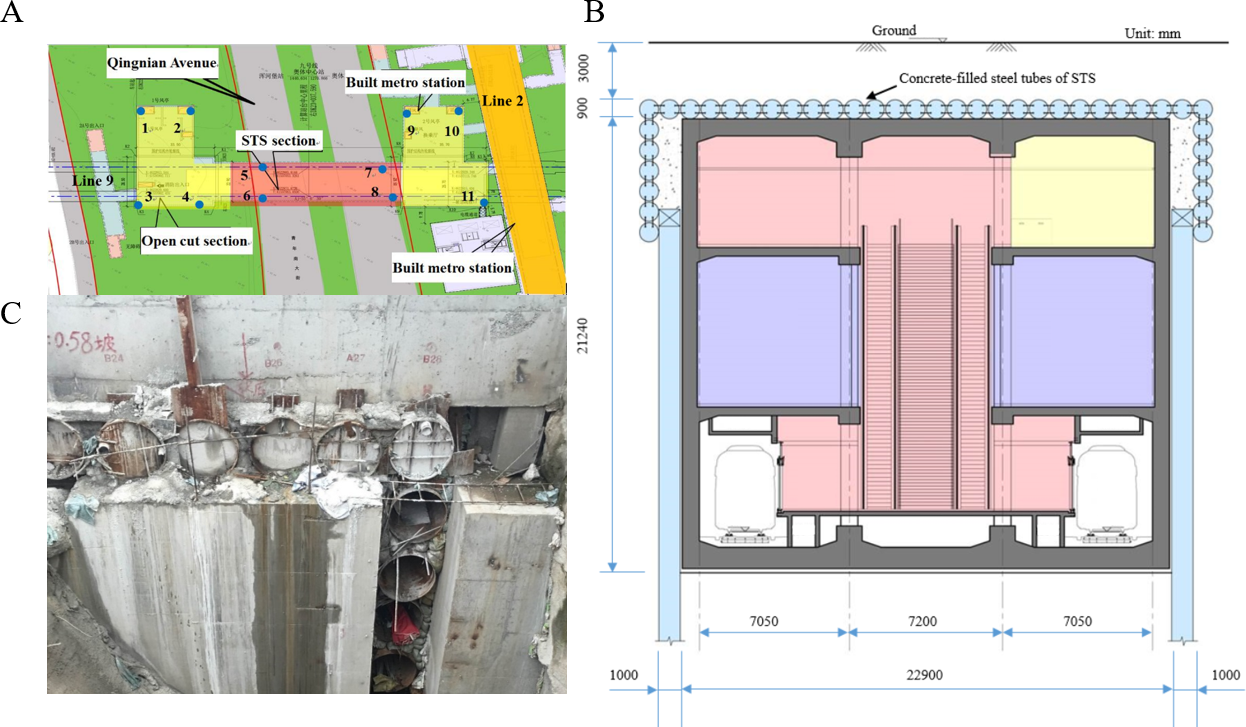
Shenyang Metro Line 9’s olympic center station. (A) Overview. (B) Cross-section. (C) STS structure concrete-filled steel tubes.

Experimental testing was carried out to validate the applicability of the proposed UCT based on the CS algorithm. The Shenyang Metro line 9’s Olympic Center station is constructed with a pipe-roofing STS structure. It is a triple-deck tunnel in a rectangular shape with a section size of approximately 22.9 m in span and 21.24 m in height and with an overburden thickness of approximately 3.0 m, as shown in Fig 8.

Each concrete-filled steel tube needs to be identified using ultrasonic inspection before the service stage to ensure the load-carrying capacity of the STS structure, which constitutes huge work. Thus, the UCT technique based on the CS algorithm is proposed to reduce the cost of inspection.

**Fig 9:**
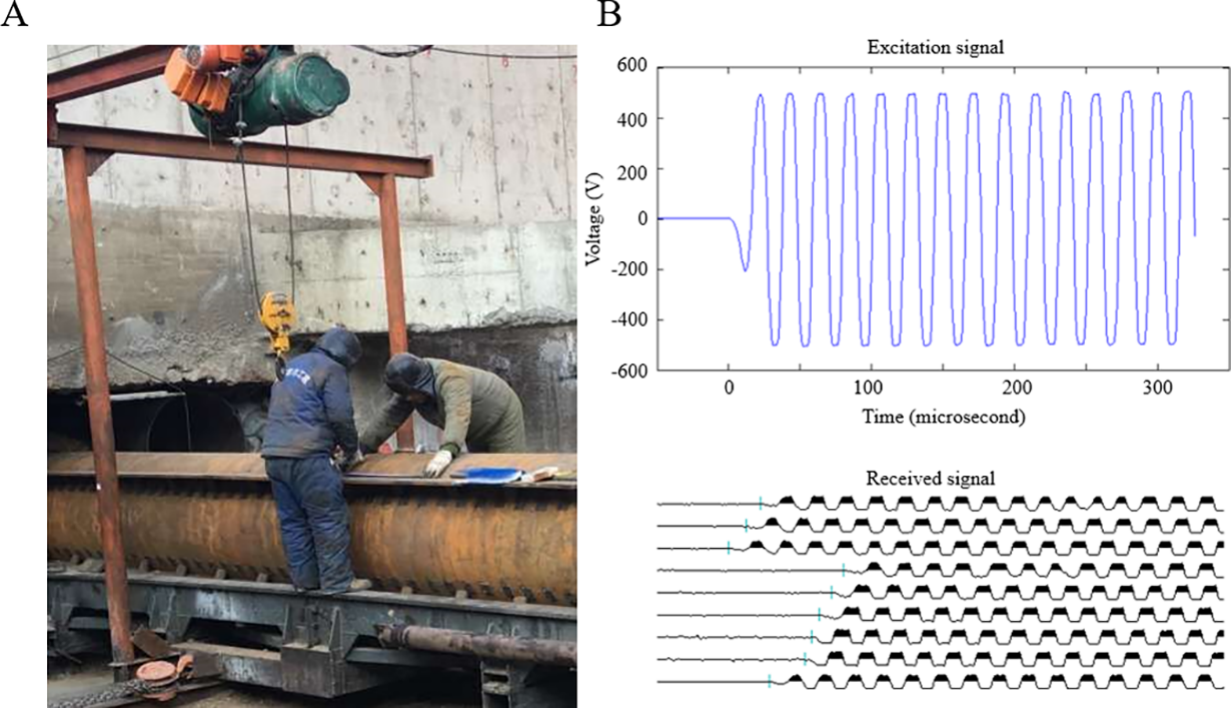
Ultrasonic inspection of a real-world STS structure concrete-filled steel tube. (A) Schematic of damage detection. (B) Excitation signal and received signals.

Ultrasonic NDT equipment, NU62 with 2 probes, was employed to detect the damage in the concrete-filled steel tubes as the transducer-receiver pair. The transducer and receiver were attached on the two ends of the selected path to launch and receive the ultrasonic waves. The transducer sends a 500 V excitation signal introducing ultrasonic waves at a central frequency of 50 kHz and with a wavelength of 0.08 m into the tubes. The receiver acquired the waveform to obtain the travel time of the ultrasonic waves propagating along the path (Fig 9). Each measurement was carried out based on the random Bernoulli distributed matrix as shown in Fig 3.

Fig 9B shows the acquired waveforms and travel time, which is the arrival time of the first wave indicated by the vertical bar. The travel times obtained in the measurement stage were assembled as the time vector ***T***_***m***_ in Eq (8) according to the order of selected paths. The slowness differences in the microstructures, Δ***s***, representing the internal situation of the tube were reconstructed by using the ℓ1-minimization algorithm as expressed by Eq (10).

**Fig 10:**
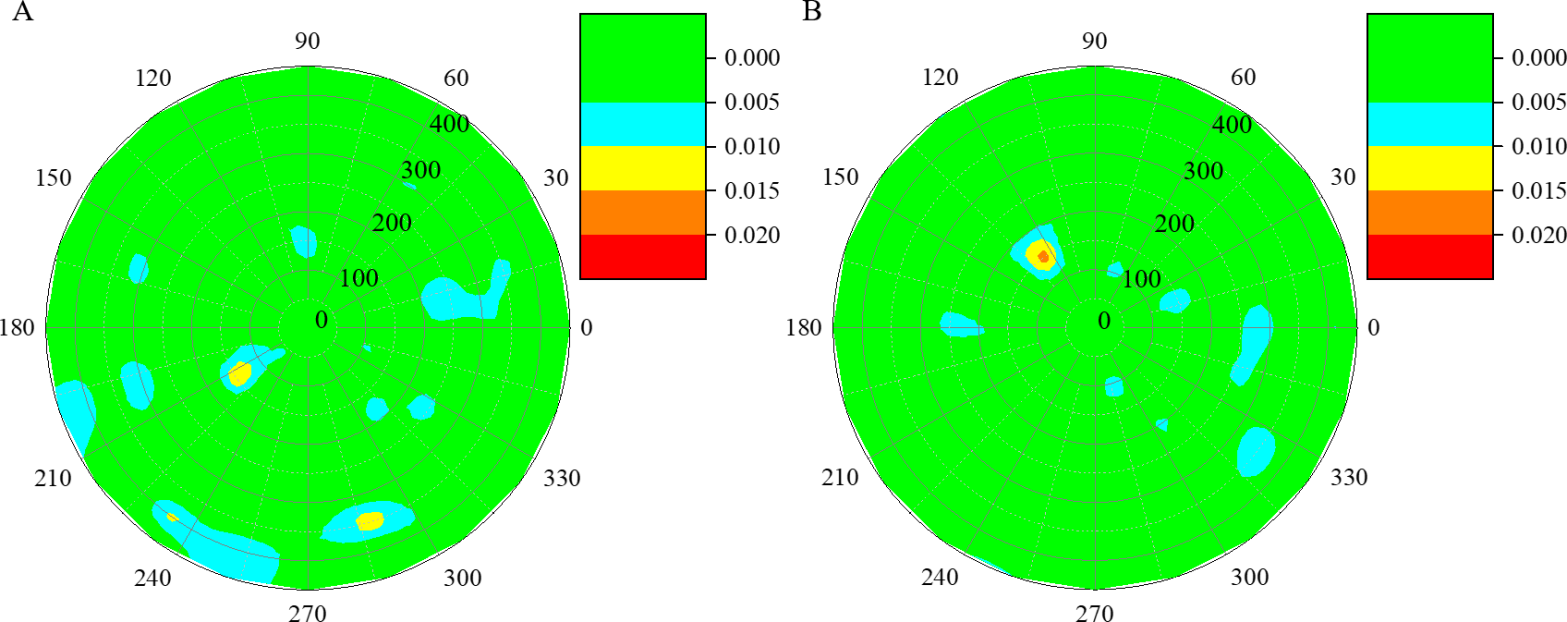
Reconstructed damage images of the concrete-filled steel tube in Shenyang Metro line 9 using the rapid UCT technique in the CS platform. (A) Cross-section 1. (B) Cross-section 2.

Reconstructed results of the real-world concrete-filled steel tube are shown in Fig 10. The first reconstructed internal situation of the tube in location 1 indicates two big holes and one small hole in this part of the tube, whose locations are about (140,210°), (400,235°), and (350,290°), respectively, as shown in Fig 10A). The reconstructed internal situation of cross section 2 is shown in Fig 10B) demonstrating only one hole at location (150,130°) in this part of the tube. Additionally, several small flaws whose slowness difference was less than 0.010 were also identified by the proposed algorithm. By using the proposed method, it is feasible to utilize considerably fewer detections than traditional UCT to obtain reasonably accurate damage detection results.

**Fig 11:**
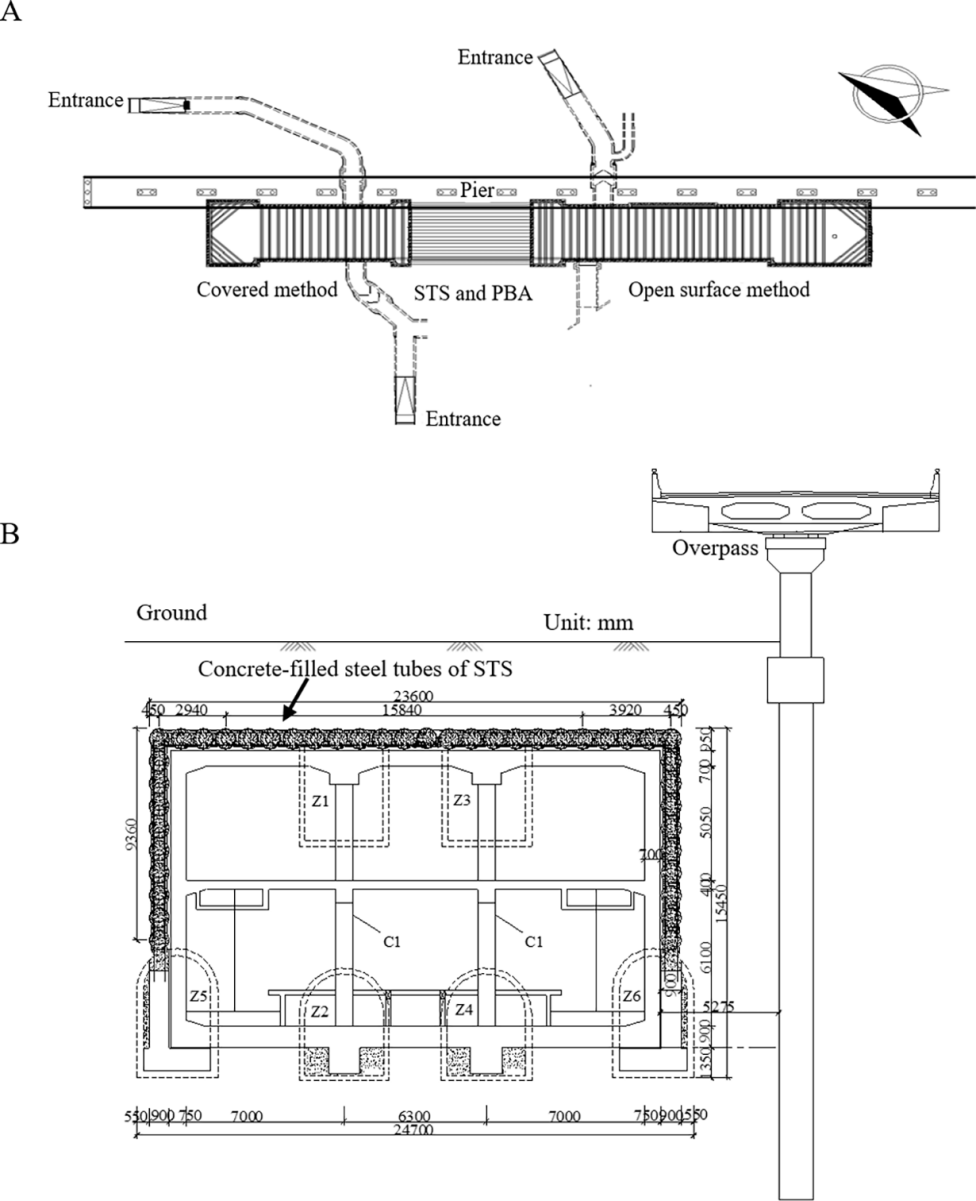
Shenyang Metro Line 10’s Northeastern street station. (A) Overview. (B) Cross-section.

The proposed method was carried on another inspection project of the Shenyang Metro line 10. Northeastern Street station is a transfer station between Shenyang Metro line 10 and line 7 (Fig 11). The total length of the station is 225.95m, the width of a standard cross section is 24.7m, and the buried depth of the bottom floor of the station is 17.5m, the overburden layer is about 3.4m. Considering the complicated traffic and intensive buildings, STS technique and PBA method were combined to construct the station. The construction is divided into three sections: (1) covered excavation used in the north of the station; (2) open surface method adopted in the south of station; (3) combined STS and Pile-Beam-Arch (PBA) method in the middle segment. It is a new challenge for the UCT inspection of the concrete-filled structures in this station.

**Fig 12:**
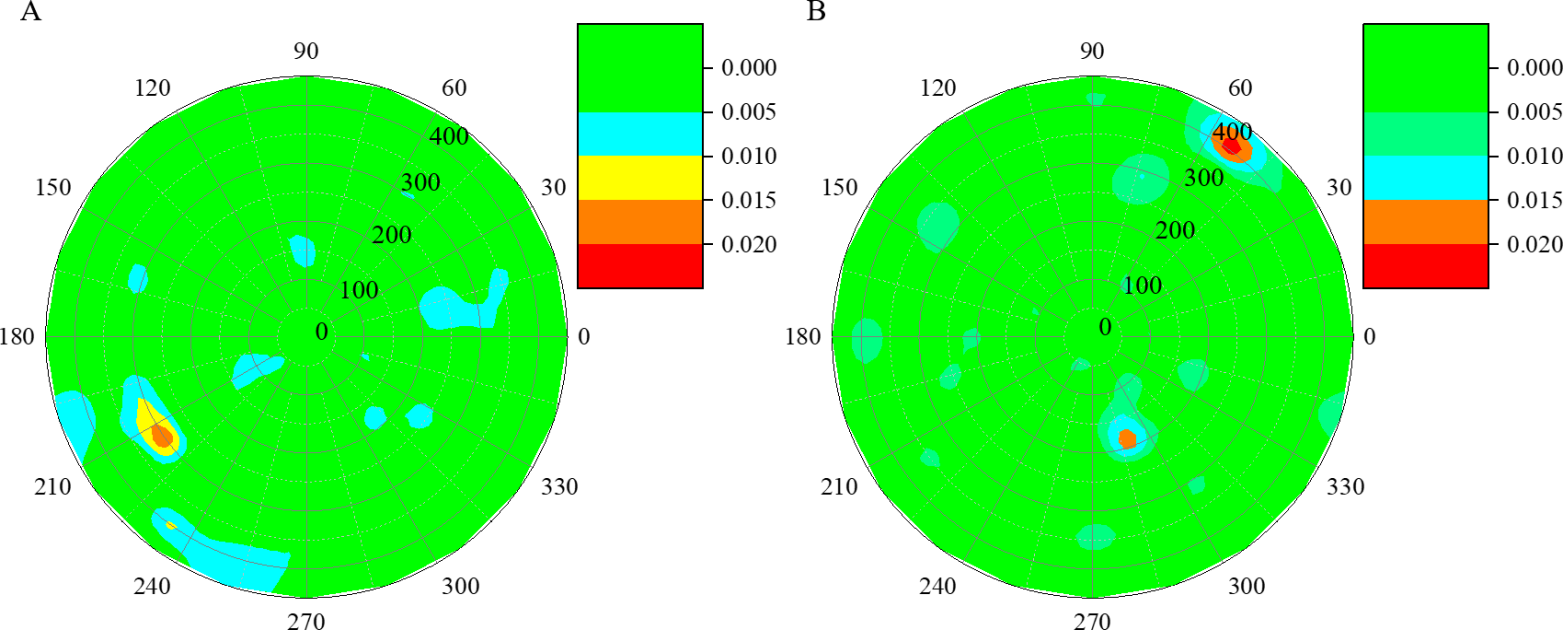
Reconstructed damage images of the concrete-filled steel tube in Shenyang Metro line 10 using the proposed rapid UCT technique. (A) Reconstructed damage images of cross-section 1. (B) Cross-section 2.

To reduce the cost of inspection procedure, the proposed method was nominated in the damage detection of the Northeastern Street station. Following the flowchart in Fig 3, the imaging results of reconstructed by the rapid UCT technique are shown in Fig 12. The proposed rapid UCT inspection technique has the capability of damage detection in the STS structure with a high level of accuracy and with fewer required measurements, which is more convenient and efficient than the traditional UCT technique.

## Conclusions

This study proposes a novel UCT reconstruction technique in the CS platform based on random low-rate measurement paths and the ℓ1-minimization algorithm. It provides an alternative to the traditional UCT damage detection technique with the benefit of low-rate measurements, rapid inspection, and high accuracy of UCT measuring and imaging.

The damage detection problem naturally meets the requirement of the CS platform because of the sparse distribution of defects in the monitored structure. Thus, the low-rate in the CS theory sampling method was applied to speed up the processing of UCT imaging. A flowchart of the rapid UCT technique was drawn to summarize the application of the ultrasonic inspection task of STS structures of the Shenyang Metro’s Olympic Center station and Northeastern Street station. Both the numerical simulation and a real-world testing of the concrete tube were carried out to validate the proposed method. The reconstructed results show the proposed method has great potential to identify the sought-after flaws and damage in an STS tube with significantly fewer measurements and with high accuracy.

In future work, the proposed method should be applied in more UCT inspections of structures and constructions, especially at the service stage. The randomly selected measurement net of the improved UCT technique should be optimized. In addition, other analytical methods, such as the variational iteration method and the homotopy perturbation method will be studied to improve future reconstruction results.
